# Cabbage (*Brassica oleracea var. capitata*) Protects against H_2_O_2_-Induced Oxidative Stress by Preventing Mitochondrial Dysfunction in H9c2 Cardiomyoblasts

**DOI:** 10.1155/2018/2179021

**Published:** 2018-08-12

**Authors:** Dong Kwon Yang

**Affiliations:** Department of Veterinary Pharmacology and Toxicology, College of Veterinary Medicine, Chonbuk National University, Iksan Campus, Iksan-si, Jeollabuk-do 54596, Republic of Korea

## Abstract

Oxidative stress plays an important role in the progression of cardiac diseases, including ischemia/reperfusion injury, myocardial infarction, and heart failure. Growing evidence indicates that cabbage has various pharmacological properties against a wide range of diseases, such as cardiovascular diseases, hepatic diseases, and cancer. However, little is known about its effects on oxidative stress in cardiomyocytes or the underlying mechanisms. Therefore, the present study examined the effects of cabbage extract on oxidative stress in H9c2 cardiomyoblasts. Cell viability, reactive oxygen species (ROS) production, apoptosis, mitochondrial functions, and expression levels of mitogen-activated protein kinase (MAPK) proteins were analyzed to elucidate the antioxidant effects of this extract. Cabbage extract protected against H_2_O_2_-induced cell death and did not elicit any cytotoxic effects. In addition, cabbage extract suppressed ROS production and increased expression of antioxidant proteins (SOD-1, catalase, and GPx). Cabbage extract also inhibited apoptotic responses and activation of MAPK proteins (ERK1/2, JNK, and p-38) in oxidative stress-exposed H9c2 cells. Notably, cabbage extract preserved mitochondrial functions upon oxidative stress. These findings reveal that cabbage extract protects against oxidative stress and suggest that it can be used as an alternative therapeutic strategy to prevent the oxidative stress in the heart.

## 1. Introduction

Oxidative stress arises due to a disturbance in the balance of oxidant and antioxidant systems in the body and is characterized by excessive reactive oxygen species (ROS) production [1]. ROS are oxygen-based chemically reactive species including superoxide (O_2_^−^), the hydroxyl radical (·OH), and hydrogen peroxide (H_2_O_2_). Among them, H_2_O_2_ is a major ROS and an important precursor of other types of ROS [2]. Excessive accumulation of ROS due to oxidative stress can lead to apoptosis, lipid peroxidation, and mitochondrial dysfunction [3]. Therefore, continued oxidative stress causes the progression and development of diseases [4, 5].

Importantly, oxidative stress plays a crucial role in the pathological progression of various heart diseases, including ischemic heart disease (IHD), also known as coronary artery disease, hypertension, and ischemia/reperfusion (I/R) injury [6–8]. Among them, IHD, which could cause myocardial infarction, is the leading cause of morbidity and mortality worldwide [9]. Until now, the effective treatment for IHD is surgical intervention. However, surgical restoration of blood flow to the ischemic region is paradoxically accompanied by cardiac injury called I/R injury [10]. Oxidative stress plays a crucial role in the pathophysiology of this I/R injury [11].

Overproduction of ROS, which mainly generated in the mitochondria, causes mitochondrial oxidative stress and further triggers mitochondrial dysfunction, including mitochondrial biogenesis, fatty acid metabolism, and antioxidant defense mechanisms. The heart, especially, as a high-energy-consuming organ, is susceptible to the mitochondrial dysfunction. Indeed, emerging evidences have linked mitochondrial dysfunction resulting from oxidative stress to many cardiac diseases, including myocardial infarction, cardiac hypertrophy, and heart failure [12]. Therefore, an antioxidant strategy should be considered as a possible approach to prevent the cardiac diseases.

Cabbage (*Brassica oleracea* var.* capitata*) from the Brassicaceae family is an important vegetable worldwide. It grows extensively in more than 90 countries, such as coastal regions of southern and western Europe [13]. Cabbage is rich in various nutrients, including calcium, proteins, and vitamins C and E and contains various bioactive compounds with pharmacological properties, such as luteolin, myricetin, quercetin, and polyphenols [14]. Consequently, cabbage has been widely used in traditional medicine to treat various diseases. Specifically, it is used to alleviate symptoms associated with gastrointestinal disorders (gastritis, peptic ulcers, and irritable bowel syndrome) and idiopathic cephalalgia as well as treat injuries [15]. Growing evidence indicates that cabbage has pharmacological activities against various diseases, including liver cirrhosis, hepatitis, cancer, and hypocholesterolemia [16–19]. A recent study demonstrated that cabbage prevents pancreatitis and identified six bioactive compounds, including several flavonoids, by gas chromatography-mass spectrometry analysis [14]. In addition, another recent study reported that white cabbage essential oil contains organic polysulphides and has antioxidant and hepatoprotective properties in rats with carbon tetrachloride-induced liver damage [20]. However, the effects of cabbage on oxidative stress in cardiac cells have not been studied.

Present study demonstrated that cabbage protects against H_2_O_2_-induced oxidative stress in H9c2 cardiomyoblast by preventing mitochondrial dysfunction.

## 2. Materials and Methods

### 2.1. Preparation of Cabbage Extract

Fresh cabbages were purchased from a local market in Korea, dried in an incubator at 60°C, and converted into a powder using an electric blender. The dried powder was extracted in 99% methanol (powder sample/99% methanol, 1:8) at 90°C for 3 h. The extracts were filtered and evaporated in a rotary evaporator (EYELA, Tokyo, Japan). The resultant yield of extract was 21.6% of dry weight. The extracts were stored in −80°C for further study.

### 2.2. Cell Culture and Induction of Oxidative Stress

H9c2 cells were obtained from the Korea Cell Line Bank (Seoul, Korea) and cultured in Dulbecco's modified Eagle's medium (Cat. No. 11995-065, GIBCO-BRL, Grand Island, NE, USA) supplemented with 10% fetal bovine serum (Cat. No. 1600004, GIBCO-BRL) and 1% antibiotics (Cat. No. 15240062, GIBCO-BRL) at 37°C in 5% CO_2_. Cabbage extracts were dissolved in 0.1% dimethyl sulfoxide (DMSO, Cat. No. D8418, Sigma, St. Louis, MO, USA) for treatment. After incubation for 24 h, cells were cultured in serum-free medium for at least 2 h, treated with 100, 200, and 300 *μ*g/ml cabbage extract for 24 h, and control cells were treated with 0.1% DMSO. 500 *μ*M H_2_O_2_ was treated for another 24 h to induce oxidative stress.

### 2.3. Cell Viability Assay

Cell viability was assessed using MTT (3-[4,5-dimethylthiazol-2-yl]-2, 5-diphenyltetrazolium Bromide; Cat. No. M2128, Sigma). Briefly, cells were seeded into 96-well plates (2000 cells/well) and treated with H_2_O_2_ alone or were pretreated with different concentrations of cabbage extract (100, 200, and 300 *μ*g/ml). Thereafter, 0.5 mg/ml MTT was added to each well. After incubation at 37°C for 2 h, the supernatants were removed and the crystals were dissolved in 100 *μ*l DMSO. Absorbance was measured at 570 nm using a spectrophotometer (Spectra Max M5; Molecular Devices, Sunnyvale, CA, USA).

### 2.4. Measurement of ROS Production

Intracellular ROS production was measured based on the fluorescence intensity of DCF-DA (2′, 7′-dichlorofluorescin-diacetate; Cat. No. D399, ThermoFisher Scientific Inc., Waltham, MA, USA). Briefly, cells in 6-well plates (1X10^5^cells/well) were treated with H_2_O_2_ alone or were pretreated with different concentrations of cabbage extract (100, 200, and 300 *μ*g/ml) and then treated with 1 *μ*M DCF-DA for 30 min at 37°C. Cells were observed under a fluorescence microscope (IX-81; Olympus Corp., Shinjuku, Tokyo, Japan). The fluorescence intensity was determined using a spectrophotometer (Spectra Max) with excitation and emission wavelengths of 488 and 515 nm, respectively.

### 2.5. Hoechst 33342 Staining

Cells were treated with H_2_O_2_ alone or were pretreated with different concentrations of cabbage extract (100, 200, and 300 *μ*g/ml), fixed with 4% paraformaldehyde for 30 min at room temperature and stained with 10 *μ*g/ml Hoechst 33342 (Cat. No. 62249, ThermoFisher Scientific Inc.) for 30 min at 37°C. The stained nuclei were observed under a fluorescence microscope (IX-81; Olympus Corp.).

### 2.6. Terminal Deoxynucleotidyl Transferase dUTP end Labeling (TUNEL) Staining

Cells were treated with H_2_O_2_ alone or were pretreated with different concentrations of cabbage extract (100, 200, and 300 *μ*g/ml) and then were fixed with 4% paraformaldehyde for 30 min at room temperature. TUNEL staining was performed using a Cell Death Detection kit (Cat. No. 11684795910, Roche Diagnostics, Manheim, Germany).

### 2.7. Mitochondrial Transmembrane Potential (MMP) Assessment

MMP was measured by staining with JC-1 (Cat. No. T3168, ThermoFisher Scientific Inc.). Briefly, cells in 6-well plates (1X10^5^cells/well) were treated with H_2_O_2_ alone or were pretreated with different concentrations of cabbage extract (100, 200, and 300 *μ*g/ml) and then incubated with 10 *μ*g/ml JC-1 for 20 min at 37°C. JC-1-labeled cells were observed under a fluorescence microscope (IX-81; Olympus Corp.). The fluorescence intensity of JC-1 was determined using a spectrophotometer (Spectra Max) with excitation and emission wavelengths of 550 nm excitation and 600 nm emission, respectively, for red fluorescence, and 485 nm excitation and 535 nm, respectively, for green fluorescence.

### 2.8. Western Blot Analysis

The cells were treated with H_2_O_2_ alone or were pretreated with different concentrations of cabbage extract (100, 200, and 300 *μ*g/ml), harvested, and lysed in RIPA buffer (1% NP-40, 50 mM Tris-HCl, pH 7.4, 150 mM NaCl, and 10 mM NaF) containing a protease inhibitor cocktail (Cat. No. 78438, ThermoFisher Scientific Inc.) and a phosphatase inhibitor cocktail (Cat. No. 4906845001, Roche Diagnostics). Protein homogenates were separated on SDS-PAGE gels and transferred to PVDF membranes (Cat. No. ISEQ00010, EMD Millipore Inc., Billerica, MA, USA). After blocking for 1 h with 5% bovine serum albumin, the membranes were incubated overnight at 4°C with antibodies against superoxide dismutase (SOD)-1 (Cat. No. sc-101523, Santa Cruz Biotechnology, Santa Cruz, CA, USA), catalase (Cat. No. #14097, Cell Signaling Tech., Danvers, MA, USA), glutathione peroxidase (Cat. No. sc-133160, GPx; Santa Cruz Biotechnology), total or phosphorylated extracellular signal-regulated kinase 1/2 (ERK 1/2; Cat. No. #9102 and #9101 for total and phospho-ERK 1/2, Cell Signaling Tech.), total or phosphorylated c-Jun N-terminal kinase (JNK; Cat. No. #9252 and #9251 for total and phospho-JNK, Cell Signaling Tech.), total or phosphorylated p38 (Cat. No. #9212 and #9211 for total and phospho-p38, Cell Signaling Tech.), Bax (Cat. No. #2772, Cell Signaling Tech.), Bcl-2 (Cat. No. sc-492, Santa Cruz Biotechnology), cleaved caspase 3 (Cat. No. #9664, Cell Signaling Tech.), and *β*-actin (Cat. No. sc-47778, Santa Cruz Biotechnology). The membranes were then incubated with the appropriate horseradish peroxidase-conjugated secondary antibodies (Jackson ImmunoResearch Lab., Inc., West Grove, PA, USA) at room temperature for 1 h. Signals were detected using an Immobilon Western Chemiluminescence kit (Cat. No. WBKLS0100, Millipore Corp., Billerica, MA, USA) and a UVITEC Mini HD9 system (Cleaver Scientific Ltd., Warwickshire, UK). The intensity of each protein band was quantified using NIH Image J software.

### 2.9. Quantitative Real-Time Polymerase Chain Reaction (qRT-PCR)

Total RNA was isolated from the cells treated with H_2_O_2_ alone or were pretreated with different concentrations of cabbage extract (100, 200, and 300 *μ*g/ml) by using a Ribospin™ II kit (Cat. No. 304-150, GeneAll Biotechnology Co., LTD, Seoul, Korea). To examine the mRNA expression levels of mitochondrial biogenesis genes, 1 *μ*g total RNA from each group of cells was reverse transcribed into cDNA using ImProm II reverse transcriptase (Cat. No. A3802, Promega Co., Madison, WI, USA) with oligo-dT priming. qRT-PCR was conducted using a TaKaRa Thermal Cycler Dice Real-Time System (Takara Bio. Inc., Shiga, Japan) with SYBR Green (Cat. No. RR420A, Takara) as a fluorescent dye. The primer sequences were as follows: peroxisome proliferator-activated receptor *α* (PPAR*α*), forward 5′-GGC AAT GCA CTG AAC ATC GAG-3′ and reverse 5′-GCC GAA TAG TTC GCC GAA AG-3′; peroxisome proliferator-activated receptor *γ* coactivator (PGC)-1*β*, forward 5′-GTG AGA TAG TCG AGT GCC AGG TG-3′ and reverse 5′-TTC TCA GGG TAG CGC CGT TC-3′; estrogen-related receptor *α* (ERR*α*), forward 5′-GCT GAA AGC TCT GGC CCT TG-3′ and reverse 5′-TGC TCC ACA GCC TCA GCA T-3′; nuclear respiratory factor (NRF)-1, forward 5′-CAC TCT GGC TGA AGC CAC CTT AC-3′ and reverse 5′-TCA CGG CTT TGC TGA TGG TC-3′; and 18S, forward 5′-TTC TGG CCA ACG GTC TAG ACA AC-3′ and reverse 5′-CCA GTG GTC TTG GTG TGC TGA-3′.

### 2.10. Statistical Analysis

Data were analyzed using a one-way analysis of variance (ANOVA) with the Bonferroni post hoc test using Prism 5.03 (GraphPad Software Inc., San Diego, CA, USA). All the results are expressed as mean ± SEM.* p* values < 0.05 were considered statistically significant.

## 3. Results

### 3.1. Cabbage Extracts Protect H9c2 Cardiomyocytes against H_*2*_O_*2*_-Induced Oxidative Stress

**Figure 1:**
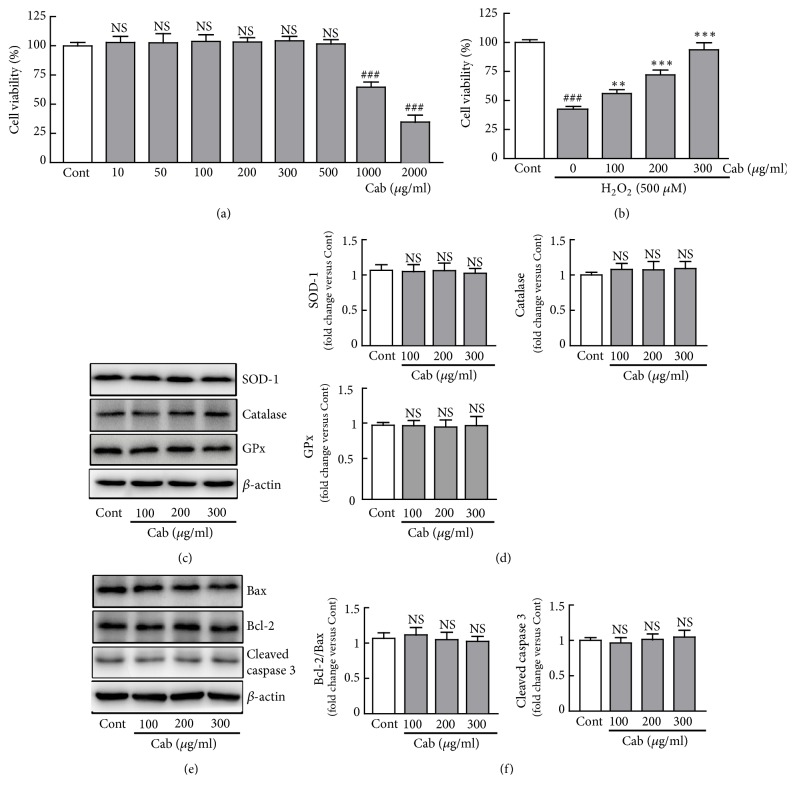
Cabbage extract protects H9c2 cardiomyocytes against H_2_O_2_-induced oxidative stress. The MTT assay was performed using (a) cells treated with 10, 50, 100, 200, 300, 500, 1000, and 2000 *μ*g/ml cabbage extract for 48 h or (b) cells pretreated with 100, 200, and 300 *μ*g/ml cabbage extract for 24 h prior to treatment with 500 *μ*M H_2_O_2_ for another 24 h. (c) Western blot analysis of SOD-1, catalase, and GPx protein expression levels in cells treated with cabbage extract for 48 h. (e) Western blot analysis of Bax, Bcl-2, and cleaved caspase 3 protein expression levels in Cu I-treated cells for 48 h. (d, f) Protein expression levels were quantified by scanning densitometry. *β*-Actin was used as the loading control. Data are expressed as the % changes ± SEM versus control cells from three independent experiments. Significance was analyzed by a one-way ANOVA followed by the Bonferroni post hoc test. ^###^P < 0.001 versus control cells. *∗∗* P < 0.01 and *∗∗∗* P < 0.001 versus H_2_O_2_ alone-treated cells. Cont, control; Cab, cabbage extract; NS, not significant.

To determine the protective effects of cabbage extract against H_2_O_2_-induced injury in H9c2 cardiomyocytes, the MTT assay was performed to assess the viability of cells treated with 10, 50, 100, 200, 300, 500, 1000, and 2000 *μ*g/ml cabbage extract for 48 h alone. The results have shown that cell viability did not significantly change in the cells treated with the concentrations from 10 to 5000 *μ*g/ml cabbage extract; otherwise, the treatments of 1000 and 2000 *μ*g/ml cabbage extract caused reduction in the cell viability compared with that in control cells (Figures 1(a) and 1(b)). In addition, the cells treated with 100 to 300 *μ*g/ml cabbage extract have shown antioxidant proteins (SOD-1, catalase, and GPx) and apoptosis-related proteins (Bax, Bcl-2, and cleaved caspase 3) (Figures 1(c)–1(f)). Therefore, 100, 200, and 300 *μ*g/ml cabbage extract were chosen to elucidate the effects against oxidative stress on H9c2 cardiomyocytes. The viability of H_2_O_2_-treated cells was reduced to 42.6% compared with that of control cells. Pretreatment with 100, 200, and 300 *μ*g/ml cabbage extract for 24 h significantly restored the viability of H_2_O_2_-treated cells to 56%, 72%, and 93%, respectively, compared with that of control cells (Figure 1(b)). These results indicate that cabbage extract prevents death of H9c2 cardiomyocytes caused by H_2_O_2_-induced oxidative stress in a dose-dependent manner.

### 3.2. Cabbage Extract Inhibits H_*2*_O_*2*_-Induced ROS Production in H9c2 Cardiomyocytes

**Figure 2:**
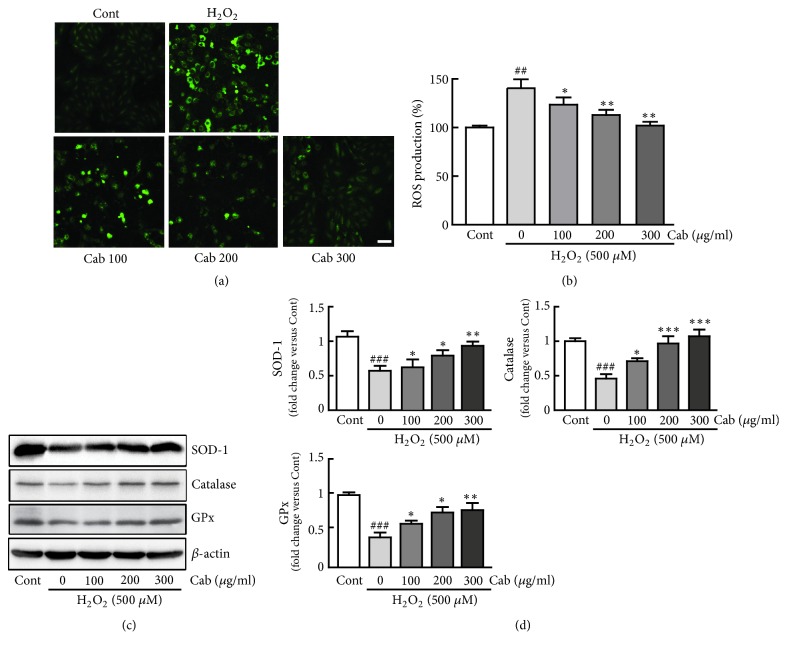
Cabbage extract inhibits H_2_O_2_-induced ROS production in H9c2 cardiomyocytes. ROS production was assessed by incubating cells with DCFH-DA for 30 min. (a) Representative images of and (b) fluorescence intensities in H9c2 cardiomyocytes treated with 500 *μ*M H_2_O_2_ for 24 h following pretreatment with 100, 200, and 300 *μ*g/ml cabbage extract for 24 h. (c) Western blot analysis of SOD-1, catalase, and GPx. (d) Protein expression levels were quantified by scanning densitometry. *β*-Actin was used as the loading control. Western blot analysis was performed in triplicate with three independent samples. Data are expressed as fold changes ± SEM versus control cells. Significance was analyzed by a one-way ANOVA followed by the Bonferroni post hoc test. ^##^ P < 0.01 and ^###^ P < 0.001 versus control cells. *∗* P < 0.05, *∗∗* P < 0.01, and *∗∗∗* P < 0.001 versus H_2_O_2_ alone-treated cells. Cont, control; Cab, cabbage extract. Scale bar, 100 *μ*m.

To determine whether cabbage extract can reduce ROS production, H9c2 cardiomyocytes were pretreated with cabbage extract (100, 200, and 300 *μ*g/ml) for 24 h and then treated with 500 *μ*M H_2_O_2_ for an additional 24 h. ROS levels were assessed using DCFH-DA. H_2_O_2_ treatment significantly increased intracellular ROS generation (140.6% versus control cells). However, pretreatment with cabbage extract dramatically decreased the ROS level in a dose-dependent manner (123.5%, 112.9%, and 102.1% in cells pretreated with 100, 200, and 300 *μ*g/ml cabbage extract versus control cells, respectively) (Figures 2(a) and 2(b)).

ROS-scavenging proteins, including SOD, catalase, and GPx, play important roles in the oxidant/antioxidant balance and prevent oxidative stress; therefore, their protein expression levels were analyzed by western blotting. H_2_O_2_ treatment significantly decreased the expression levels of these proteins in a dose-dependent manner (0.53-, 0.45-, and 0.42-fold decreases in SOD1, catalase, and GPx expression versus control cells, respectively) (Figures 2(c) and 2(d)). As expected, pretreatment with cabbage extract dramatically restored these protein expression levels in a dose-dependent manner compared with those in H_2_O_2_ alone-treated cells. Hence, cabbage extract effectively inhibits ROS production and restores expression of antioxidant proteins in H_2_O_2_-treated H9c2 cells.

### 3.3. Cabbage Extract Blocks the MAPK Signaling Pathway in H_*2*_O_*2*_-Treated H9c2 Cardiomyocytes

**Figure 3:**
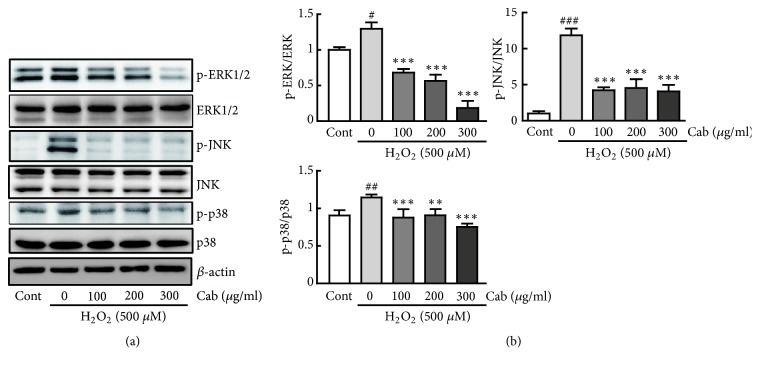
Cabbage extract blocks the MAPK signaling pathway in H_2_O_2_-treated H9c2 cardiomyocytes. (a) Western blot analysis of the protein expression levels of the total and phosphorylated forms of ERK1/2, JNK, and p38 in H9c2 cardiomyocytes treated with 500 *μ*M H_2_O_2_ for 24 h following pretreatment with 100, 200, and 300 *μ*g/ml cabbage extract for 24 h. (b) The protein expression levels were quantified by scanning densitometry. *β*-Actin was used as the loading control. Western blot analysis was performed in triplicate with three independent samples. Data are expressed as fold changes ± SEM versus control cells. Significance was analyzed using a one-way ANOVA followed by the Bonferroni post hoc test. ^#^ P < 0.05, ^##^ P < 0.01, and ^###^ P < 0.001 versus control cells. *∗∗* P < 0.01 and *∗∗∗* P < 0.001 versus H_2_O_2_ alone-treated cells. Cont, control; Cab, cabbage extract.

To identify the molecular mechanisms underlying the protective effects of cabbage extract against oxidative stress in H9c2 cells, the protein expression levels of MAPKs (ERK1/2, JNK, and p38), which are involved in proapoptotic signaling pathways upon oxidative stress, were determined. Western blot analysis revealed that phosphorylation of ERK1/2, JNK, and p38 was significantly higher in H_2_O_2_ alone-treated cells than in control cells (1.3-, 11.8-, and 1.27-fold increases in p-ERK1/2/ERK1/2, p-JNK/JNK, and p-p38/p38 versus control cells, respectively) (Figure 3). By contrast, pretreatment with cabbage extract followed by treatment with 500 *μ*M H_2_O_2_ inhibited phosphorylation of all these proteins in H9c2 cells in a dose-dependent manner (47.3%, 56.4%, and 85.7% decreases in p-ERK1/2/ERK1/2; 64.4%, 61.7%, and 65.6% decreases in p-JNK/JNK; and 23.4%, 20.7%, and 34.2% decreases in p-p38/p38 in cells pretreated with 100, 200, and 300 *μ*g/ml cabbage extract versus H_2_O_2_ alone-treated cells, respectively) (Figure 3). These data suggest that cabbage extract inhibits the MAPK signaling pathway in oxidative stress-exposed H9c2 cells.

### 3.4. Cabbage Extract Suppresses H_*2*_O_*2*_-Induced Apoptosis in H9c2 Cardiomyocytes

**Figure 4:**
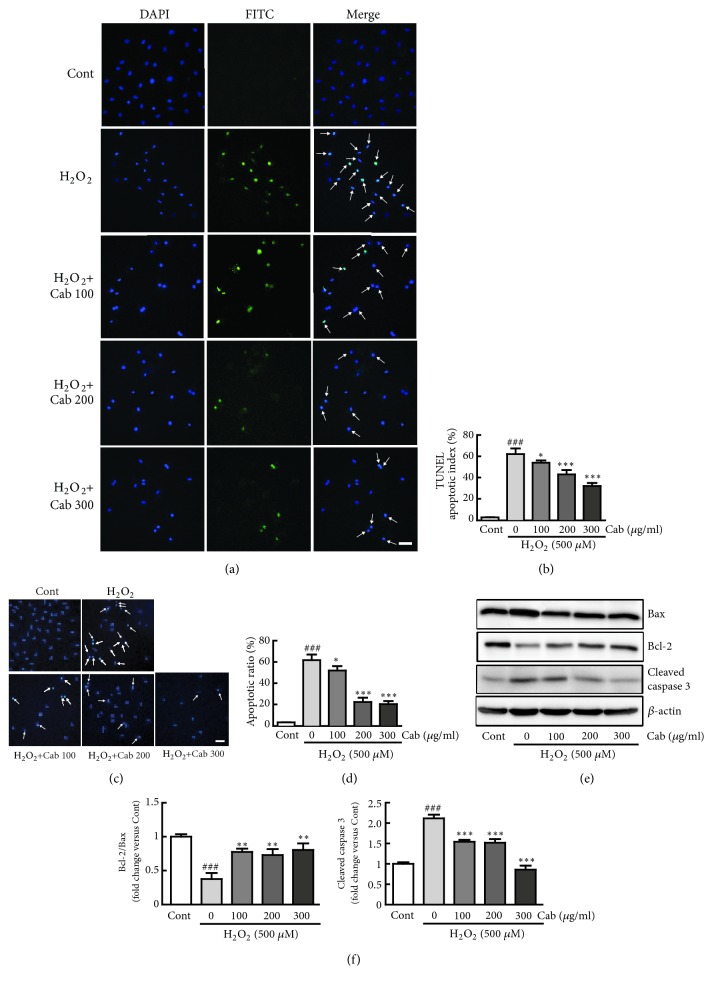
Cabbage extract suppresses H_2_O_2_-induced apoptosis in H9c2 cardiomyocytes. Apoptosis was determined by TUNEL, Hoechst staining, and western blot analysis of apoptosis-related proteins. Representative images of H9c2 cardiomyocytes treated with 500 *μ*M H_2_O_2_ for 24 h following pretreatment with 100, 200, and 300 *μ*g/ml cabbage extract for 24 h in the (a) TUNEL and (c) Hoechst assays. (b) The apoptotic index was calculated by determining the percentage of (b) TUNEL-positive or (d) Hoechst-positive cells. (e) Western blot analysis of the protein expression levels of Bax, Bcl-2, and cleaved caspase 3. (f) The protein expression levels were quantified by scanning densitometry. *β*-Actin was used as the loading control. Western blot analysis was performed in triplicate with three independent samples. Data are expressed as fold changes ± SEM versus control cells. Significance was analyzed by a one-way ANOVA followed by the Bonferroni post hoc test. ^###^ P < 0.001 versus control cells. *∗* P < 0.05, *∗∗* P < 0.01, and *∗∗∗* P < 0.001 versus H_2_O_2_ alone-treated cells. Cont, control; Cab, cabbage extract. Scale bar, 100 *μ*m.

To evaluate the effects of cabbage extract on H_2_O_2_-induced apoptosis in H9c2 cells, apoptotic cells were examined by TUNEL and Hoechst 33342 staining. The percentage of TUNEL-positive cells was much higher among H_2_O_2_ alone-treated cells than among control cells (green; 62% increase in TUNEL-positive cells versus control cells) (Figures 4(a) and 4(b)). Meanwhile, the percentage of TUNEL-positive cells was significantly lower among cells pretreated with 100, 200, and 300 *μ*g/ml cabbage extract than H_2_O_2_ alone-treated cells (12.9%, 30.6%, and 48.4% decreases in TUNEL-positive cells versus H_2_O_2_ alone-treated cells, respectively) (Figures 4(a) and 4(b)). Hoechst staining showed that the percentage of apoptotic cells was much higher among H_2_O_2_ alone-treated cells than among control cells (61.5% increase versus control cells). The percentage of apoptotic cells was lower among cells pretreated with cabbage extract than among H_2_O_2_ alone-treated cells (15.8%, 63.9%, and 67.2% decreases in cells pretreated with 100, 200, and 300 *μ*g/ml cabbage extract versus H_2_O_2_ alone-treated cells, respectively) (Figures 4(c) and 4(d)). To further evaluate the antiapoptotic effects of cabbage extract, the protein levels of the apoptosis regulators Bax, Bcl-2, and cleaved caspase 3 were determined. The levels of Bax and cleaved caspase 3 were significantly upregulated in H_2_O_2_-treated cells, but these increases were prevented by pretreatment with cabbage extract. Conversely, Bcl-2 expression was significantly increased in cells pretreated with cabbage extracts but decreased in H_2_O_2_ alone-treated cells (106.4%, 94.2%, and 114.0% increases in Bcl-2/Bax and 27.2%, 28.2%, and 59.4% decreases in cleaved caspase 3 in cells pretreated with 100, 200, and 300 *μ*g/ml cabbage extract versus H_2_O_2_ alone-treated cells, respectively) (Figures 4(e) and 4(f)).

### 3.5. Cabbage Extract Prevents Mitochondrial Dysfunction upon H_*2*_O_*2*_-Induced Oxidative Stress in H9c2 Cardiomyocytes

**Figure 5:**
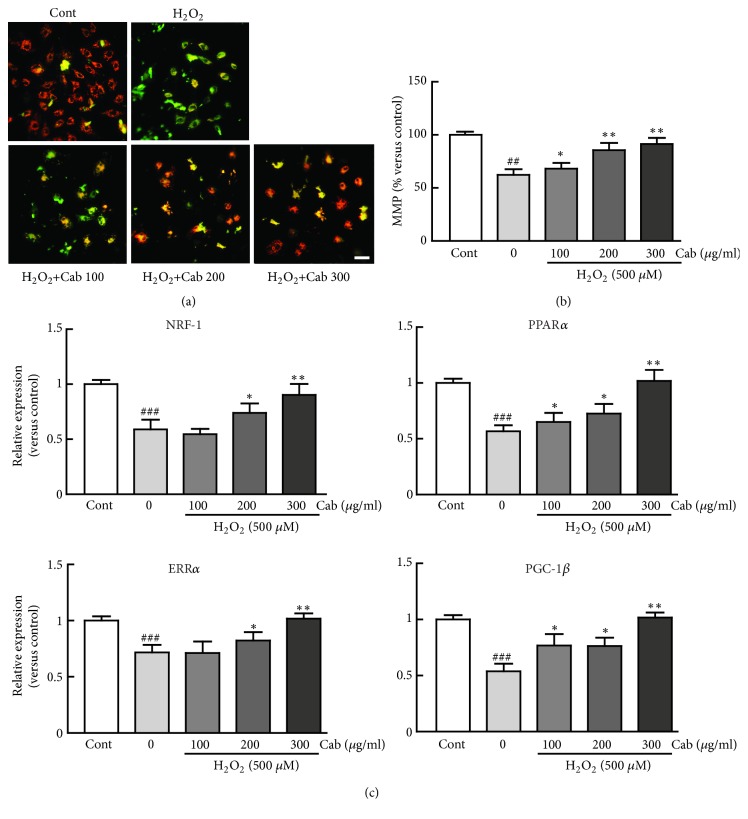
Cabbage extract prevents mitochondrial dysfunction upon H_2_O_2_-induced oxidative stress in H9c2 cardiomyocytes. The MMP was determined by incubating cells with JC-1 for 20 min. (a) Representative images of and (b) fluorescence intensities in H9c2 cardiomyocytes treated with 500 *μ*M H_2_O_2_ for 24 h following pretreatment with 100, 200, and 300 *μ*g/ml cabbage extract for 24 h. (c) Quantitative RT-PCR analysis of mitochondrial biogenesis-related genes (NRF-1, PPAR*α*, ERR*α*, and PGC-1*β*). Western blot analysis was performed in triplicate with three independent samples. Data are expressed as fold changes ± SEM versus control cells. Significance was analyzed by a one-way ANOVA followed by the Bonferroni post hoc test. ^##^ P < 0.01 and ^###^ P < 0.001 versus control cells. *∗* P < 0.05 and *∗∗* P < 0.01 versus H_2_O_2_ alone-treated cells. Cont, control; Cab, cabbage extract. Scale bar, 100 *μ*m.

To assess the prevention of oxidative stress by cabbage extract, the MMP and mitochondrial integrity in H_2_O_2_-treated H9c2 cells were analyzed. The percentage of cells labeled with the MMP sensor JC-1 was dramatically lower among H_2_O_2_ alone-treated cells than among control cells (62.3% decrease among H_2_O_2_-treated cells versus control cells) (Figures 5(a) and 5(b)). As expected, the percentage of JC-1 labeled cells was significantly higher among cells pretreated with 100, 200, and 300 *μ*g/ml cabbage extract than among H_2_O_2_ alone-treated cells (14.3%, 37.4%, and 46.7% increases versus H_2_O_2_ alone-treated cells, respectively) (Figures 5(a) and 5(b)). The expression levels of genes involved in mitochondrial biogenesis, including NRF-1, PPAR*α*, ERR*α*, and PGC-1*β* were determined. The expression levels of these genes were significantly reduced in H_2_O_2_-treated cells, otherwise, and were restored by pretreatment with cabbage extract (Figure 5(c)).

## 4. Discussion

Over the past several decades, experimental and clinical studies have implicated oxidative stress mediated by excessive ROS in a variety of cardiomyopathies, such as I/R injury, myocardial infarction, and heart failure [3, 21, 22]. The heart is susceptible to oxidative stress because it contains lower levels of antioxidant proteins than other organs [23]. This decreased antioxidant capacity of cardiac cells is a major contributor to oxidative stress, which causes progression of pathological heart conditions. Therefore, a therapeutic strategy that can prevent oxidative stress in the heart is to supply exogenous antioxidants or to upregulate endogenous antioxidants. Intensive efforts have been made to identify exogenous antioxidants that can prevent oxidative stress in the heart and to elucidate their underlying mechanisms. Recently, the antioxidant activities of naturally occurring, plant-derived natural compounds have been intensively studied due to their safety and efficacy against oxidative stress-induced cardiovascular diseases [24]. Various herbal plants have been identified as new agents in the treatment of oxidative stress [25–28]. Furthermore, several natural antioxidants, including vitamin E, flavonoids, and polyphenols, have been exploited [29]. Curcumin has been intensively investigated due to its potent antioxidant properties against oxidative stress in the heart as well as its inhibitory effects on various heart diseases [30, 31]. The present study sought to determine the antioxidant effects of cabbage extract in H_2_O_2_-exposed H9c2 cardiomyoblasts.

Since H_2_O_2_ causes oxidative stress and thereby markedly decreases viability and induces apoptosis in H9c2 cells [32, 33], this study confirmed that the viability of H9c2 cells was decreased following exposure to H_2_O_2_ for 24 h. However, pretreatment with cabbage extract increased the viability of H_2_O_2_-treated cells in a dose-dependent manner. Furthermore, pretreatment with cabbage extract inhibited intracellular ROS production and increased expression of antioxidant proteins, including SOD-1, catalase, and GPx, while H_2_O_2_ treatment resulted in accumulation of ROS and reduced levels of these antioxidant proteins in H9c2 cells. These results suggest that cabbage extract has protective effects against oxidative stress in H_2_O_2_-treated H9c2 cells.

Accumulation of ROS can be a crucial mediator of apoptotic cell death [34]. Apoptosis of cardiac cells, which do not proliferate once differentiated, markedly triggers heart dysfunction [35, 36]. Therefore, this study determined whether cabbage extract could prevent apoptotic responses and the related signaling pathway in H_2_O_2_-treated cells. TUNEL and Hoechst 33342 staining revealed that H_2_O_2_ treatment triggered fragmentation and condensation of DNA in nuclei, typical features of apoptosis, whereas pretreatment with cabbage extract significantly attenuated these apoptotic changes in H_2_O_2_-treated cells. In addition, cabbage extract reduced levels of proapoptotic proteins, such as Bax and cleaved caspase 3, and increased the level of Bcl-2, an antiapoptotic protein.

The MAPK signaling pathways play crucial roles in the regulation of cell survival, apoptosis, and inflammatory responses in various pathological conditions [37, 38]. Activation of these MAPK signaling pathways upon oxidative stress can stimulate apoptosis [21, 39]. Therefore, the present study analyzed the expression of three major MAPK proteins (ERK1/2, JNK, and p38) to elucidate the preventive effects of cabbage extract against oxidative stress in H9c2 cells. Here, I demonstrated that activation of these proteins by H_2_O_2_ treatment was significantly suppressed by pretreatment with cabbage extract.

Importantly, previous study demonstrated that the heart has a high-energy demand and mitochondria therefore play a pivotal role in maintaining heart functions, such as energy production and cardiac contractile function [40]. Furthermore, overproduction of ROS due to oxidative stress can damage mitochondria, and, in turn, mitochondrial dysfunction can mediate apoptosis and is one of the major contributors to cardiac diseases [41]. Thus, prevention of mitochondrial dysfunction may be a therapeutic strategy to inhibit cardiac injury caused by oxidative stress. Regarding this, I sought to elucidate the preventive effects of cabbage extract against mitochondrial dysfunction upon oxidative stress for the underlying mechanism. As expected, pretreatment with cabbage extract dramatically restored the mitochondrial function against oxidative stress by performing several mitochondrial functional assays, including MMP assay, and expression of mitochondrial biogenesis genes.

This study provides new evidence that cabbage extract protects against oxidative stress in H9c2 cardiomyoblast by inhibiting ROS production and apoptosis and by preserving mitochondrial functions. Additionally, the present study demonstrates that cabbage suppresses activation of proapoptotic MAPK proteins in H9c2 cells exposed to oxidative stress. I propose that cabbage is a potential antioxidant-agent to protect against oxidative stress.
